# Tunnel face stability in strata rich in water with soft upper and hard lower layers

**DOI:** 10.1038/s41598-025-89107-9

**Published:** 2025-02-08

**Authors:** Tao Zhan, Ziwei Hu, Yan Kuang, Zhiliang Zhu, Kaiyi Liao

**Affiliations:** 1 Metro Project Management Branch of Nanchang Rail Transit Group Co., Ltd., Nanchang, China; 2 School of Civil Engineering, Central South University, Changsha, 410075 China; 3 China Construction Third Engineering Bureau Group Co., Ltd., Wuhan, China

**Keywords:** Tunnel face stability, Soft upper and hard lower strata, Pore water pressure, Limit analysis, Natural hazards, Solid Earth sciences

## Abstract

When a shield tunnel is excavated in water-rich strata with soft upper and hard lower layers, the failure mode of the tunnel face may shift from an overall failure mode to a partial failure mode. To address this issue, three-dimensional discrete failure mechanisms for both partial and overall failure modes are established on the basis of the upper bound theorem and spatial discretization techniques. The influence of pore water pressure is also considered, leading to the development of a method for calculating the critical support force of tunnel faces in such strata, while taking into account both failure modes and the effects of groundwater. Through parameter analysis, factors such as the tunnel diameter, proportion of soft soil, stratum cohesion, and pore water pressure coefficient significantly influence the tunnel face failure mode. A comprehensive critical support force, which takes both partial and overall failure modes into account, is proposed. The parameter analysis reveals that this comprehensive critical support force exhibits complex variations under the influence of multiple parameters. At the same time, a method is proposed to determine the upper and lower bounds of the ultimate support force, based on the calculation results under no seepage and free seepage conditions at the excavation face. The entire method provides a valuable reference for the stability analysis of tunnel faces in water-rich strata with soft upper and hard lower layers.

## Introduction

With the rapid development of urban rail transit, the strata through which metro tunnels pass have become increasingly complex^1–3^. Although the shield method can effectively reduce the risks of surface settlement and collapse, disturbances to the surrounding soil during excavation are unavoidable^4,5^. The stability of the tunnel face during shield excavation is crucial for ensuring the safety of shield tunnelling. Many incidents of surface collapse during urban underground tunnel construction have been caused by tunnel face instability and insufficient support, such as the road surface collapse at Shahe Station on Guangzhou Metro Line 1 in 2019, water leakage and surface collapse on Hangzhou Metro Line 5 in 2019, water ingress and surface collapse on Foshan Metro Line 2 in 2018, and road surface collapse caused by construction in a certain section of Nanchang Metro in 2018, among others^6–8^. Engineering practices have shown that shield tunnel faces are more prone to active failure, where insufficient support force leads to soil collapse^9,10^. As a result, numerous studies have been conducted on the active failure modes and stability of shield tunnel faces via model tests, numerical simulations, and theoretical analyses^11–15^. Among these methods, theoretical analysis allows for large-scale parametric analysis, and the limit analysis method is an effective approach for assessing the stability of shield tunnel faces^16,17^.

Mollon et al.’s^18^ three-dimensional helical collapse mechanism, which is based on spatial discretization techniques, considers the entire circular failure mechanism and has been widely adopted by many researchers^19,20^. Li et al.^21^ studied the influence of pore water pressure on shield tunnel face stability and provided design charts for engineering applications. Di et al.^22^ investigated the stability of shield tunnel faces under seepage in saturated foundations, particularly for subsea tunnels, whereas Zou et al.^23^ incorporated arching effects to develop an improved failure model for the stability of bonded-frictional soil tunnel faces. Tu et al.^24^ analysed the stability of tunnel faces in inclined layered soils, validated their results using numerical methods, and provided design charts.

However, existing studies have primarily focused on overall failure modes that encompass the entire shield tunnel face, establishing failure mechanisms on the basis of this approach. When the tunnel face is located in composite strata, it is crucial to consider the transition from an overall failure mode affecting the entire cross-section to a partial failure mode occurring only in the weak strata^17,25^. Some studies have addressed the establishment of partial failure mechanisms in non-water-bearing strata^26–29^. For example, Senent and Jimenez^26^ used numerical simulations and limit analysis to study the transition of failure mechanisms in layered soils under dry conditions, reporting that when the upper soil layer is relatively weak, the failure mode shifts from overall failure to partial failure. Ding et al.^27^ derived the critical support pressure for partial failure in shield tunnel faces within soft upper and hard lower composite strata using the limit equilibrium method under dry conditions. Wang et al.^28^ used numerical simulation methods and limit analysis to study the partial collapse mechanism of a horseshoe-shaped tunnel face in layered soils, analysing in detail the effects of cohesion and friction angle on the transition of failure mechanisms. Ma et al.^29^ used numerical methods to analyse the critical support pressure of shield tunnel faces in soft-hard mixed strata. These studies have laid the foundation for establishing partial failure mechanisms in soft upper and hard lower composite strata. However, the influence of groundwater in water-rich strata cannot be overlooked, and further research is needed to comprehensively account for the combined effects of composite strata and groundwater.

To address this issue, the present study investigates both the overall and partial failure modes of tunnel faces caused by soft upper and hard lower strata, utilizing spatial discretization techniques to establish corresponding three-dimensional helical collapse mechanisms. On this basis, the influence of pore water pressure in water-rich strata is considered, and the relationships between parameters and the transitions between partial and overall failure modes are explored. A comprehensive critical support force that accounts for both partial and overall failures is proposed, and a limit support force range considering different levels of seepage is proposed, providing a theoretical basis for engineering applications.

## Overall and partial failure modes of tunnel faces and their mechanisms

### Problem statement

In water-rich conditions, the stability of the tunnel face during shield tunnelling in soft upper and hard lower strata is quite complex. When the strength difference between the upper and lower strata is small, instability and collapse may occur across the entire tunnel face. However, when there is a significant strength difference between the strata, the lower hard stratum can maintain its stability, and the instability and collapse will only occur in the upper stratum. One key point when using limit analysis for calculations is the construction of a reasonable failure mechanism. If the actual failure mode involves partial collapse in the upper soft soil but an overall failure mechanism covering the entire tunnel face is used for the calculation, the stability of the tunnel face may be overestimated, leading to a failure in the design method. The research problem addressed in this study is to propose a limit analysis method for evaluating tunnel face stability in water-rich conditions with soft upper and hard lower strata. By considering both local and overall failure mechanisms, the method can better assess the stability of the tunnel face under these conditions.

### Overall and partial failure modes

**Fig. 1:**
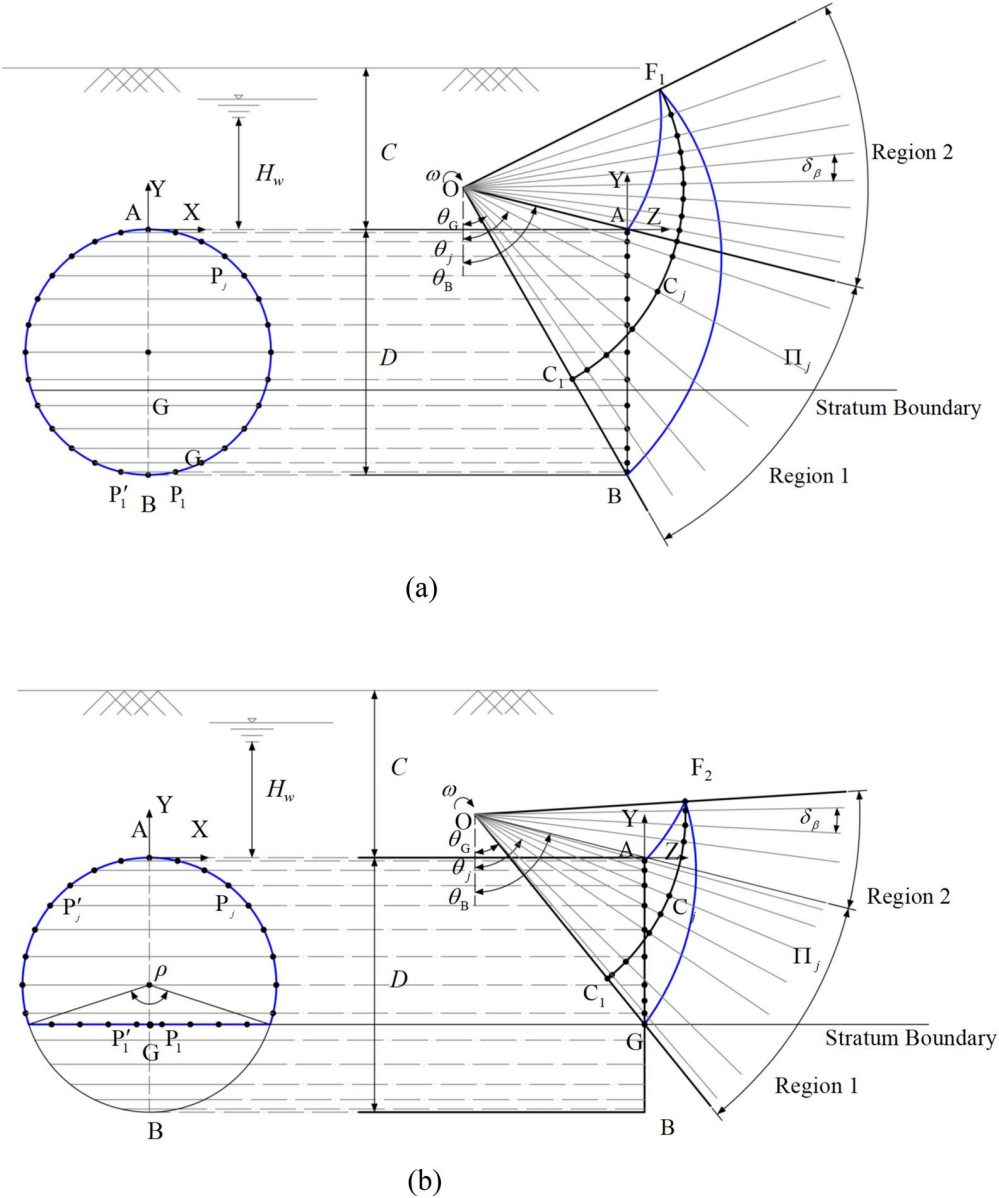
Overall and partial failure modes. (**a**) Overall failure mode, (**b**) Partial failure mode.

On the basis of relevant studies^26–29^, this paper considers two failure modes: the overall failure mode that covers the entire tunnel face and the partial failure mode that covers the soft upper stratum. Figure 1 illustrates the failure mechanisms corresponding to the overall and partial failure modes of a shield tunnel face in soft upper and hard lower strata. In the figure, the tunnel diameter is denoted by *D*, the global coordinate origin is denoted by A, and the rotation center of the failure mechanism is denoted by O, where *ρ* represents the angle occupied by the hard soil layer. The entire failure mechanism is assumed to rotate clockwise around point O with an angular velocity *ω*. For both the overall failure mode and the partial failure mode, the contour lines of the failure mechanism at the shield tunnel face are circular and arch shaped, respectively. The upper and lower boundaries of the symmetry plane in the overall failure mechanism are denoted by AF and BF, whereas the upper boundaries in the partial failure mechanism are AF and GF, all of which are logarithmic spiral curves. Considering the presence of two strata, the BF is represented as a segmented logarithmic spiral curve. The equations for the upper and lower boundaries can be expressed as:

1 $$ \left\{ {\begin{array}{*{20}l} {{\text{AF}}:r_{{\text{A}}} = r_{{{\text{OA}}}} \cdot {\text{e}}^{{(\theta - \theta_{{\text{A}}} )\tan \varphi_{{\text{U}}} }} } \hfill \\ {{\text{BF}}:\left\{ {\begin{array}{*{20}l} {r_{{\text{B}}} = r_{{{\text{OB}}}} \cdot {\text{e}}^{{(\theta_{{\text{B}}} - \theta )\tan \varphi_{{\text{L}}} }} ,\theta \le \theta_{{\text{G}}} } \hfill \\ {r_{B} = r_{OB} \cdot e^{{(\theta_{{\text{B}}} - \theta_{{\text{G}}} )\tan \varphi_{{\text{L}}} + (\theta_{{\text{G}}} - \theta )\tan \varphi_{{\text{U}}} }} ,\theta > \theta_{G} } \hfill \\ \end{array} } \right.} \hfill \\ {{\text{GF}}:r_{{\text{G}}} = r_{{{\text{OG}}}} \cdot {\text{e}}^{{(\theta - \theta_{{\text{G}}} )\tan \varphi_{{_{{\text{U}}} }} }} } \hfill \\ \end{array} } \right. $$

where $r_{{{\text{OA}}}}$, $r_{{{\text{OB}}}}$ and $r_{{{\text{OG}}}}$ are the lengths of OA, OB, and OG, respectively, where $\theta_{{\text{A}}}$, $\theta_{{\text{B}}}$, and $\theta_{{\text{G}}}$ are the angles between OA, OB, and OG, and the vertical line, respectively. where $\varphi_{L}$ and $\varphi_{U}$ are the friction angles of the lower hard strata and the upper soft strata, respectively. The lines AF and BF intersect at F_1_, and AF and GF intersect at F_2_. The angles corresponding to F_1_ and F_2_ are given by:

2 $$ \left\{ {\begin{array}{*{20}l} {\theta_{{{\text{F}}_{{1}} }} = \frac{1}{2}\left[ {\left( {\theta_{{\text{A}}} + \theta_{{\text{B}}} } \right) - \frac{{\tan \varphi_{l} }}{{\tan \varphi_{u} }}(\theta_{{\text{G}}} + \theta_{{\text{B}}} ) - \frac{1}{{\tan \varphi_{u} }}\ln \left( {\frac{{\sin \theta_{{\text{B}}} }}{{\sin \theta_{{\text{A}}} }}} \right)} \right]} \hfill \\ {\theta_{{{\text{F}}_{2} }} = \frac{1}{2}\left[ {\left( {\theta_{{\text{A}}} + \theta_{{\text{B}}} } \right) - \frac{1}{{\tan \varphi_{u} }}\ln \left( {\frac{{\sin \theta_{{\text{B}}} }}{{\sin \theta_{{\text{A}}} }}} \right)} \right]} \hfill \\ \end{array} } \right. $$

### Spatial discretization of failure mechanisms

On the basis of the spatial discretization technique proposed by Mollon^18,30^, the principles for constructing failure mechanisms are as follows:

For the overall failure mode, 2*n* discrete points are first generated on a circular cross-section symmetrically about the Y-axis, denoted as ${\text{P}}_{j}$ and ${\text{P}}_{j}^{\prime }$, as shown in (Fig. 1a). For the partial failure mode, 2 m discrete points are generated along the arc section, and 2* k* discrete points are generated along the straight section, ensuring that the spacing between points is approximately consistent, as illustrated in (Fig. 1b).

Subsequently, radial planes in region 1 are generated. These planes are determined by the rotation center O and the discrete points ${\text{P}}_{j}$ and ${\text{P}}_{j}^{\prime }$. The radial planes in region 2 are generated by rotating the adjacent radial planes around the X-axis by a fixed angle $\delta_{\beta }$ in the counter clockwise direction. For convenience in subsequent derivations, a partial coordinate system is established in each radial plane. The center of the partial coordinate system ${\text{C}}_{j}$ is determined by the intersection of a circle with O as the center and radii OF_1_ and OF_2_ with each radial plane. The coordinates of ${\text{C}}_{j}$ in the global coordinate system are as follows:

3 $$ \left\{ {\begin{array}{*{20}l} {X_{c,j} = 0} \hfill \\ {Y_{c,j} = Y_{0} - r_{{\text{F}}} \cos \theta_{j} } \hfill \\ {Z_{c,j} = Z_{0} + r_{{\text{F}}} \cos \theta_{j} } \hfill \\ \end{array} } \right. $$

where $\theta_{j}$ is the angle between each radial plane and the negative direction of the Y-axis. For the overall failure mode and partial failure mode, where A_1_ and A_2_ represent the lengths OF_1_ and OF_2_.

**Fig. 2:**
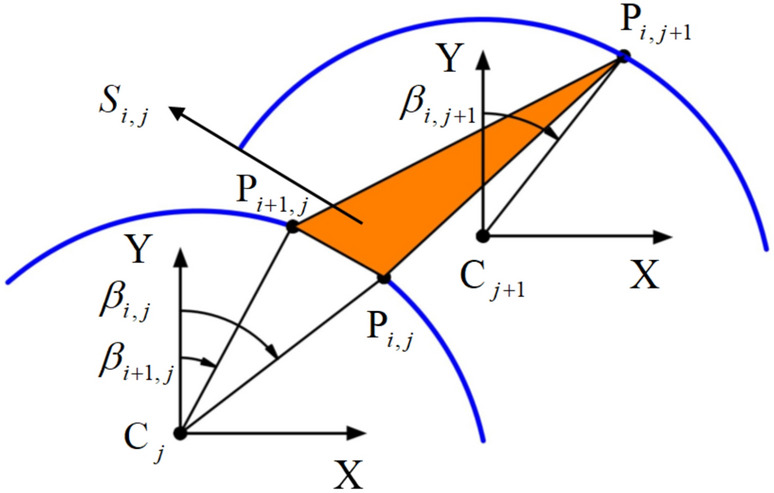
Principle of point-to-point generation.

The point-to-point generation mechanism in the failure mechanism is shown in (Fig. 2). To facilitate the representation of points on each radial plane, ${\text{P}}_{i,j}$ denotes the *i*-th point on the *j*-th radial plane. According to the point-to-point discretization mechanism, point ${\text{P}}_{i,j + 1}$ is generated by points ${\text{P}}_{i,j}$ and ${\text{P}}_{i + 1,j}$, forming a differential triangle $S_{i,j}$.

The generation of each point must satisfy three principles:

Since the generation mechanism in this study operates within the framework of the Mohr‒Coulomb criterion and associated flow laws, the normal vector of the differential triangle $S_{i,j}$ must form an angle ${{\varphi + {\uppi }} \mathord{\left/ {\vphantom {{\varphi + {\uppi }} 2}} \right. \kern-0pt} 2}$ with the velocity vector of the radial plane $\Pi_{j}$.
According to the definition of the discrete points, point ${\text{P}}_{i,j + 1}$ must lie on the radial plane $\Pi_{j + 1}$.
3). To ensure the uniformity of the discretized points, the angles corresponding to ${\text{P}}_{i,j}$, ${\text{P}}_{i + 1,j}$, and ${\text{P}}_{i,j + 1}$ within their respective radial planes must satisfy the relationship ${\text{P}}_{i,j + 1} = \left( {{\text{P}}_{i,j} + {\text{P}}_{i + 1,j} } \right)/2$.

Thus, the construction of the failure mechanism for the shield tunnel working face can be completed. The accuracy of the mechanism is determined by the number of discrete points, 2*n* (with 2 m + 2* k* for partial failure), and the discrete angle $\delta_{j}$. On the basis of existing research and preliminary calculations, the use of a total of 200 discrete points and a discrete angle of 0.5° strikes a balance between computational efficiency and accuracy.

## Analysis of the limit analysis upper bound method for tunnel face stability

Within the framework of the limit upper bound analysis theorem, the failure mechanism is considered a rigid body subjected to gravity, support pressure, pore water pressure, and soil cohesion. When the sum of the work done by external forces on the failure mechanism equals zero, failure of the mechanism occurs^31,32^. Therefore, there exists a support force such that the sum of the work done by all the external forces on the failure mechanism is exactly zero. At this point, the failure mechanism is in a critical state of impending failure, and this support pressure is termed the critical support pressure. In engineering practice, the support force employed must exceed the critical support pressure to ensure the stability of the shield tunnel face.

### Gravitational power

**Fig. 3:**
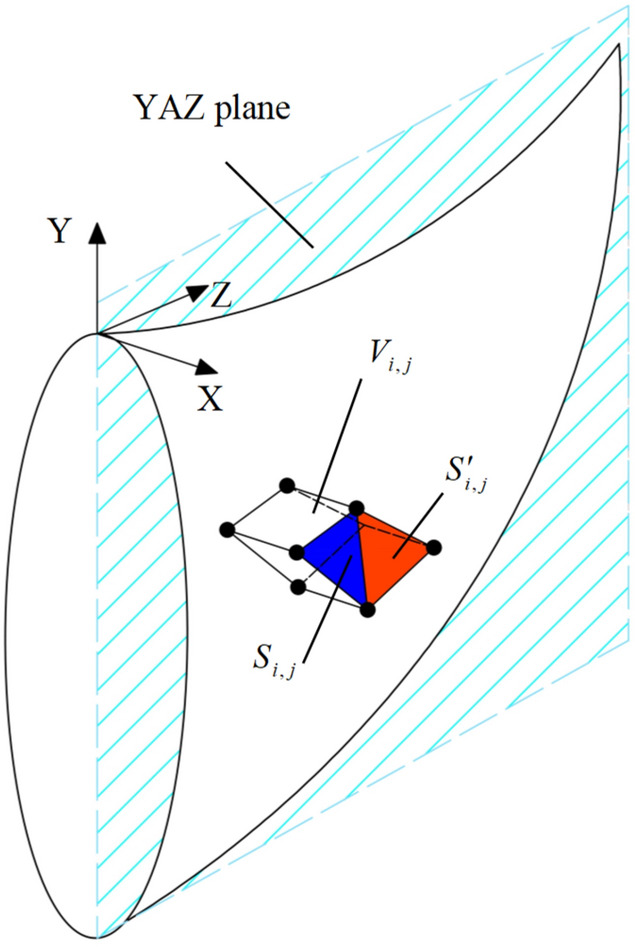
Triangular infinitesimal elements and triangular prism infinitesimal elements.

As shown in Fig. 3, adjacent discrete points form differential equilateral triangles $S_{i,j}$ and $S^{\prime}_{i,j}$. These differential triangles are projected onto the symmetry plane of the mechanism, creating a prismatic volume element $V_{i,j}$ from the projection of the quadrilateral formed by the differential triangles. Considering that the strata are water rich, the saturated unit weight is used for calculating the gravitational power. Thus, the work done by gravity on the failure mechanism can be expressed as the sum of the work done by gravity on all differential volume elements:

4 $$ W_{G} { = }\iiint_{V} {\overrightarrow {\gamma }_{sat} \cdot \overrightarrow {v} dv}{ = }\omega \gamma \sum\limits_{i,j} {\left( {R_{i,j} \cdot V_{i,j} \cdot \sin \theta_{i,j} } \right)} $$

where $V_{i,j}$ is the volume of the differential prismatic element, $\gamma_{sat}$ is the saturated unit weight of the soil, $R_{i,j}$ is the distance from the centroid of the differential volume element $V_{i,j}$ to point O, and $\theta_{i,j}$ is the angle between the line connecting O and the negative direction of the Y-axis.

### Energy dissipation

During rotational failure of the failure mechanism, the angle between the direction of the cohesion pressure and the velocity vector is always greater than 90°, meaning that cohesion always performs negative work. The work done by the cohesion force on the failure mechanism is defined for all differential area elements. The internal energy dissipation of the entire mechanism is then given by:

5 $$ W_{D} { = }\iint_{S} {c \cdot v_{i,j} } \cdot \cos \varphi {\text{dS = }}\omega c\cos \varphi \sum\limits_{i} {\sum\limits_{j} {\left( {R_{i,j} \cdot S_{i,j} + R_{i,j}{\prime} \cdot S_{i,j}{\prime} } \right)} } $$

where $S_{i,j}$ and $S^{\prime}_{i,j}$ are the areas of the positive and negative triangular elements, respectively (as shown in (Fig. 3). $R_{i,j}$ and $R^{\prime}_{i,j}$ are the distances from the centroids of the differential triangles to point O, and $\varphi$ is the friction angle of the soil.

### Power of support pressure

**Fig. 4:**
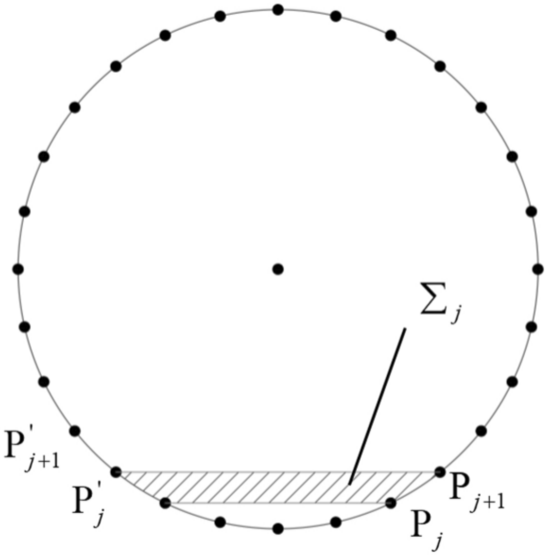
Schematic diagram of an infinitesimal element for support pressure power calculation.

When the support force is calculated, a strip element $\Sigma_{j}$ is formed by the discrete points ${\text{P}}_{j}$, ${\text{P}}_{j + 1}$, ${\text{P}}_{j}^{\prime }$, and ${\text{P}}_{j + 1}^{\prime }$ on the excavation face, as shown in (Fig. 4). Assuming that the support pressure on the excavation face is uniformly distributed across the entire shield tunnel face, the power of the support pressure on the excavation face is given by:

6 $$ W_{\sigma } { = }\iint_{\Sigma } {\overrightarrow {\sigma } \cdot \overrightarrow {v} }{\text{d}}\Sigma { = }\omega \sigma \sum\limits_{j} {\left( {R_{j} \Sigma_{j} \cos \theta_{j} } \right)} $$

where $\sigma$ is the magnitude of the support pressure, $R_{j}$ is the distance from the centroid of element $\Sigma_{j}$ to point O, and $\theta_{j}$ is the angle between the line connecting O and the centroid and the negative direction of the Y-axis.

### Pore water pressure power

For water-bearing soil layers, when the tunnel face is excavated, seepage occurs at the tunnel face because the water pressure at the tunnel face is lower than the water pressure in the soil ahead of the tunnel face^33,34^. To account for the effects of seepage, the pore water pressure in the soil needs to be determined. For the problem of seepage at the tunnel face, Li et al.^21^ based on the Laplace seepage equation, proposed a method for calculating the seepage field at the tunnel face, which can quickly compute the pore water pressure field ahead of the velocity surface. This method is used in the present study to calculate the pore water pressure in the soil ahead of the tunnel face under seepage conditions. The water head at any point is given by:

$$ \begin{gathered} h_{i} (y,z) = \sum\limits_{n = 1}^{\infty } {A_{n} } \sin \left( {\sqrt {\lambda_{n} } y} \right)\sinh \left[ {\sqrt {\lambda_{n} } z} \right] + \sum\limits_{n = 1}^{\infty } {B_{n} } \sin \left( {\sqrt {\lambda_{n} } y} \right)\sinh \left[ {\sqrt {\lambda_{n} } (h_{0} - z)} \right] \hfill \\ + \sum\limits_{n = 1}^{\infty } {C_{n} } \sin \left[ {\frac{L}{{h_{0} }}\sqrt {\lambda_{n} } z} \right]\sinh \left[ {\frac{L}{{h_{0} }}\sqrt {\lambda_{n} } y} \right] + \sum\limits_{n = 1}^{\infty } {D_{n} } \sin \left[ {\frac{L}{{h_{0} }}\sqrt {\lambda_{n} } z} \right]\sinh \left[ {\frac{L}{{h_{0} }}\sqrt {\lambda_{n} } (L - y)} \right] \hfill \\ \end{gathered} $$

where *h*_0_ is the distance from the water level to the tunnel bottom. and the specific definitions of the other parameters can be found in the study by Li et al.^21^. The pore water pressure at any point can be calculated as:

7 $$ u = \gamma_{w} \cdot h_{i} (y,z) $$

where $\gamma_{w}$ is the unit weight of water, and *z* is the distance from the calculation point to the water level.

The work done by pore water pressure on the soil can be divided into the work done to cause soil expansion and the work done on the velocity boundary of the soil. However, within the framework of the upper bound analysis, the failure mechanism is regarded as a rigid body, meaning that no soil expansion occurs. Additionally, as seepage at the working face is not considered, the pore water pressure only works at the velocity discontinuity surface of the failure mechanism, and its power is given by:

8 $$ W_{w} = \iint_{S} {u \cdot \overrightarrow {{n_{i} }} } \cdot \overrightarrow {{v_{i} }} {\text{d}}S = \omega \sum\limits_{i} {\sum\limits_{j} {\left( {u_{i,j} R_{j} \sin \varphi_{i,j} S_{i,j} } \right) + } } \omega \sum\limits_{j} {\left( {u_{j} R_{j} \Sigma_{j} \sin \varphi_{j} } \right)} $$

In the equation, the first and second terms represent the power of the pore water pressure on the velocity discontinuity surface of the failure mechanism and on the shield tunnel excavation face, respectively. $u_{i,j}$ and $u_{j}$ are the pore water pressures at the centroids of the differential elements $S_{i,j}$ and $\Sigma_{j}$, respectively.

### Ultimate support pressure

Finally, on the basis of the power balance principle of the limit upper bound method, the formula for calculating the ultimate support pressure required to maintain the stability of the shield tunnel face is obtained:

9 $$ \sigma_{t} = \frac{{W_{G} + W_{w} - W_{D} }}{{\sum\limits_{j} {\left( {R_{j} \Sigma_{j} \cos \theta_{j} } \right)} }} $$

Since the position of the rotation center O affects the ultimate support pressure, the maximum critical support pressure can be obtained via the nonlinear optimization function Fminsearch in MATLAB.

**Fig. 5:**
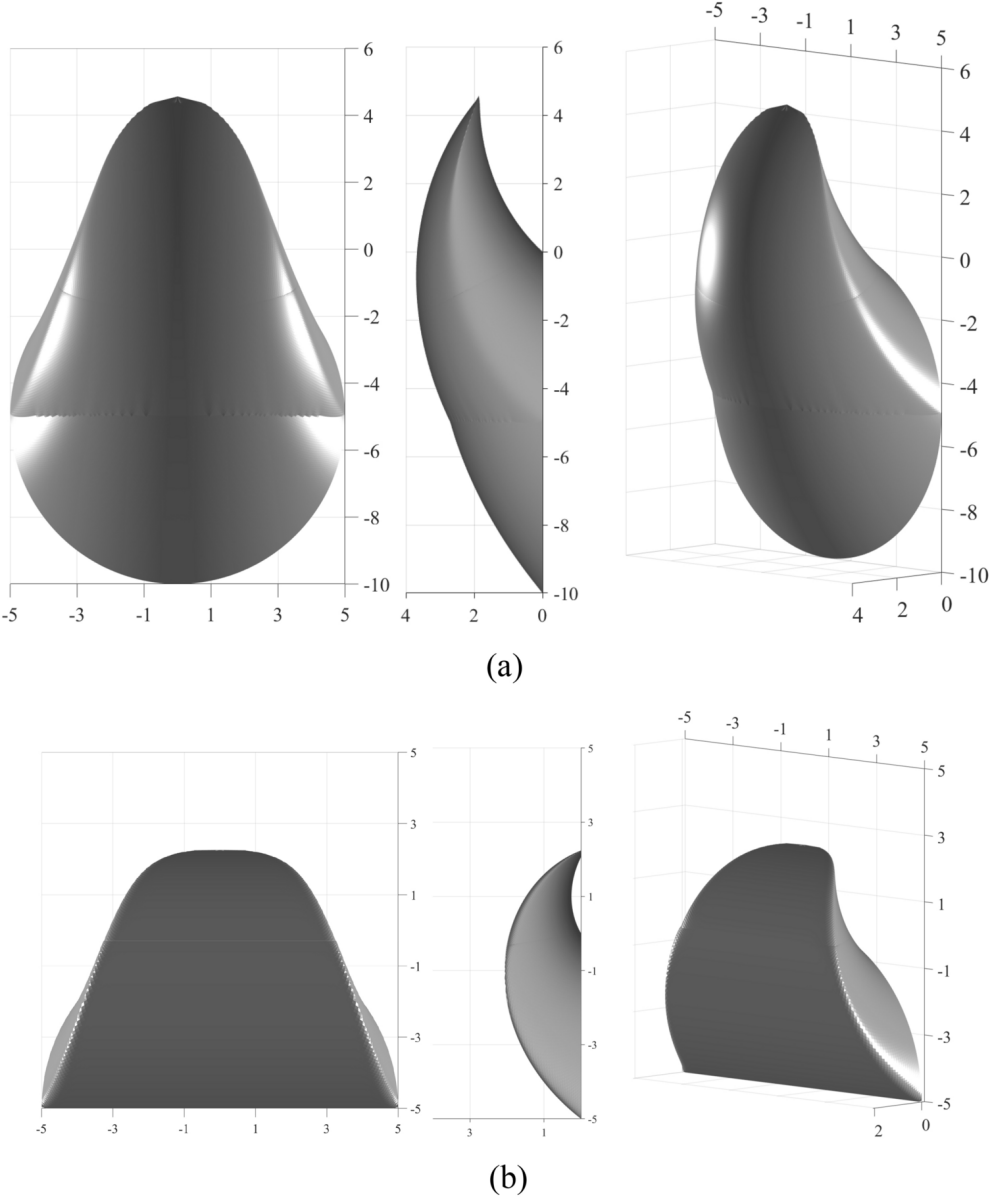
Failure mechanisms of overall and partial failure modes. Created using MATLAB (Version 2021b, https://www.mathworks.com/products/matlab.html). (**a**) Failure mechanism of the overall failure mode, (**b**) Failure mechanism of the partial failure mode.

Figure 5 shows the difference in failure mechanism shapes between the overall failure mode and partial failure mode, with the following calculation parameters: $\rho$ = 180°, $D$ = 10 m, $c_{l}$ = 20 kPa, $c_{u}$ = 5 kPa, $\varphi_{l}$ = 30°, $\varphi_{u}$ = 20°, $H_{w}$ = 10 m, and $\gamma_{sat,l}$ = $\gamma_{sat,u}$ = 20 kN/m^3^.

## Verification of the calculation model

### Degradation overall failure model considering pore water pressure

Since few studies have simultaneously considered partial failure and pore water pressure, this paper compares the proposed model with an overall failure model considering pore water pressure and a partial failure model under dry conditions to verify the applicability of the proposed model to water-rich strata and partial failure.

First, to verify the accuracy of the critical support pressure calculation considering the impact of pore water pressure, the proposed model is simplified into a model for calculating the critical support pressure of a shield tunnel excavation face in a single-layer stratum under pore water pressure without considering the effects of partial failure and stratum layering.

**Fig. 6:**
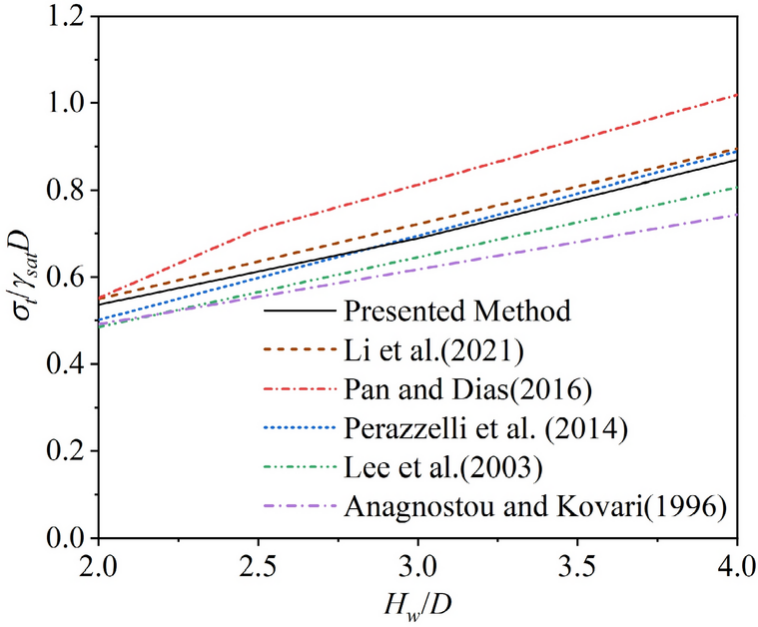
Comparison between the present study and other studies.

The calculation results of the proposed method are compared with existing studies, as shown in (Fig. 6). It can be observed that the results of this study are very close to those of Li et al.^21^ and Perazzelli et al.^35^, but slightly smaller than those of Pan and Dias^36^. This is because the method used in this study for calculating pore water pressure is based on the analytical approach proposed by Li et al., which results in a slightly smaller pore water pressure compared to the numerical results of Pan and Dias. The head distribution approximation used by Perazzelli et al. is also closer to the analytical solution of Li et al. Additionally, the results of this study are higher than those of earlier, more simplified models, such as those by Anagnostou and Kovari^37^ and Lee et al.^38^.

### Degradation partial and overall failure models in dry strata

**Fig. 7:**
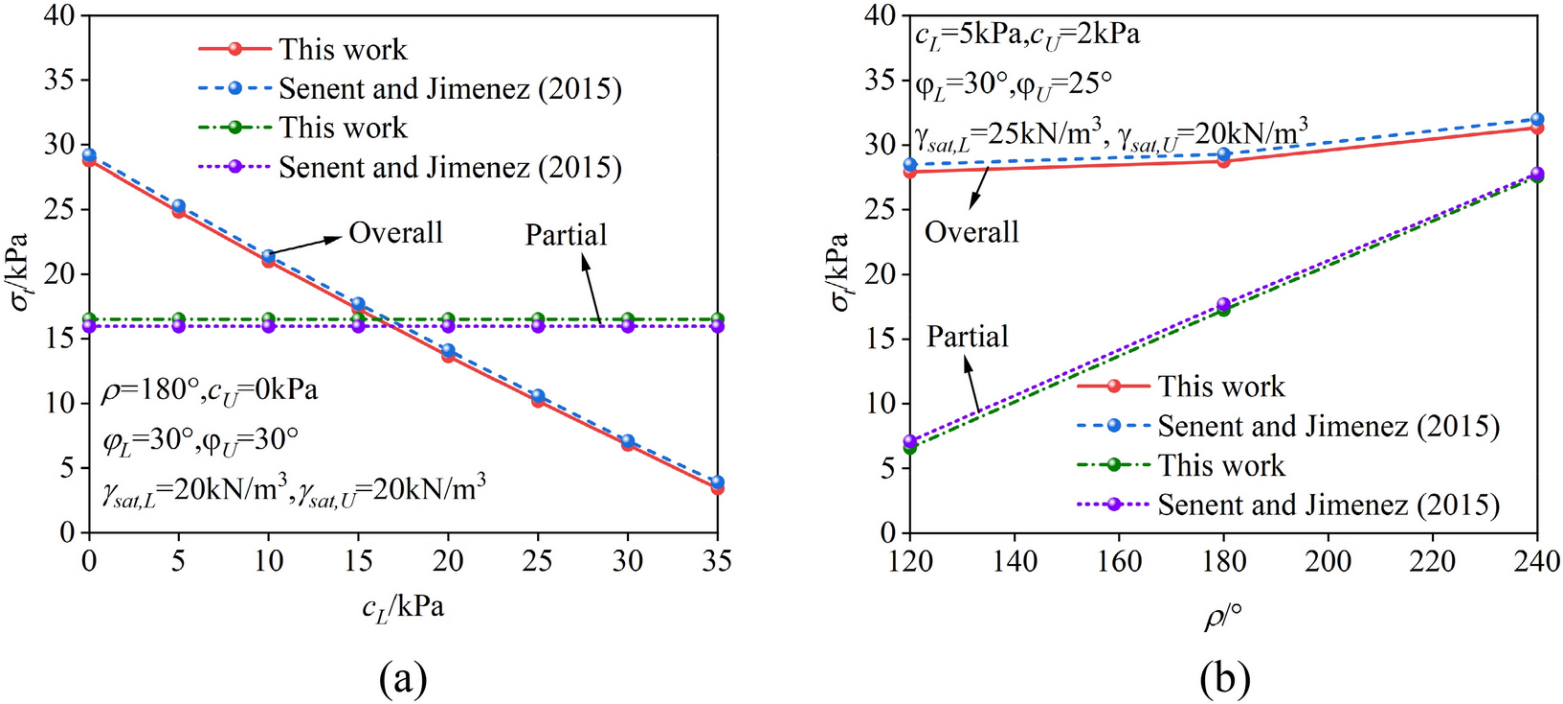
Comparison between the present study and the method of Senent and Jimenez^26^. (**a**) case1, (**b**)case2.

To verify the reliability of the proposed method for calculating the critical support pressure of the excavation face in the soft upper and hard lower strata under both overall and partial failure modes, the effects of the pore water pressure were ignored. This simplified the model into one for calculating the critical support pressure of the excavation face under dry conditions for both overall failure and partial failure. The degraded model was then compared with the results from Senent and Jimenez^26^, as shown in (Fig. 7). The analysis indicates that, in both failure modes, the solution obtained in this study is very close to that of Senent and Jimenez^26^. The deviations between the two results arise from two aspects: Senent and Jimenez^26^ applied some smoothing to the partial failure mode, whereas this study considered the entire upper stratum; additionally, the number of discrete points used in the failure mechanisms of this study and Senent and Jimenez^26^ may differ, leading to variations in the results. However, the overall deviation is small, with relative deviations of only 1% and 3% for the overall failure mode and partial failure mode, respectively, which fully meet engineering accuracy requirements. From the above analysis, it is evident that the method proposed in this study has sufficient reliability.

### Numerical simulation

**Fig. 8:**
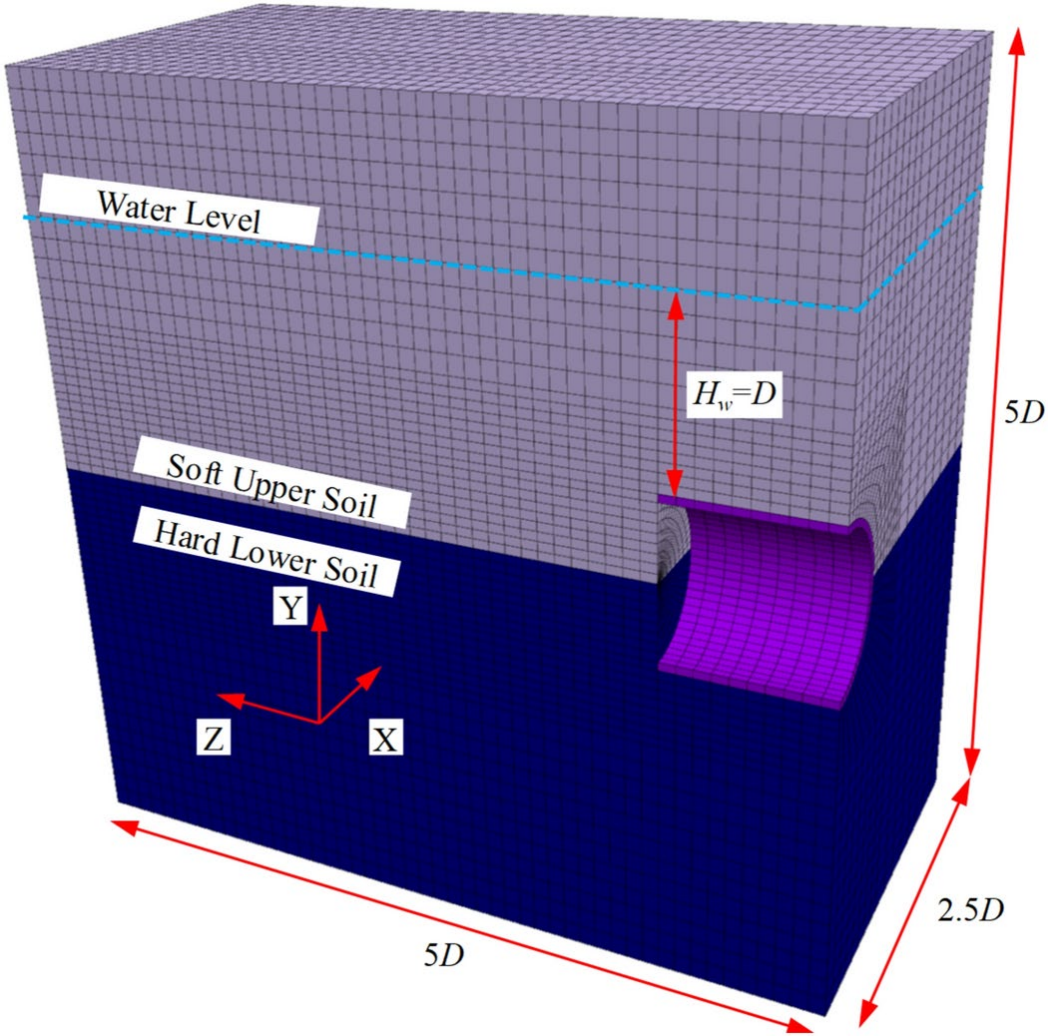
Numerical Model. Created using FLAC3D (Version 6.0, https://www.itascacg.com/software/flac3d).

Since the comparison with other studies is based on the degraded model of this study, numerical simulations were conducted to further verify the reliability of the proposed method and to compare it with traditional numerical simulation methods. A numerical model for analysing the stability of the tunnel face, as shown in the Fig. 8, was established. Considering the symmetry of the tunnel face stability problem, a half-model was used to improve analysis efficiency. Normal constraints were applied on the symmetry plane and other side surfaces, while fixed constraints were applied at the bottom of the model.

The analysis process is as follows: first, the in-situ stress balance is calculated. After the calculation is completed, the fluid calculation mode is activated to compute the seepage field in front of the tunnel face. Once the seepage field is determined, the mechanical calculation is carried out. By varying the support pressure at the tunnel face, the relationship between the maximum horizontal displacement and the support pressure is obtained. According to relevant studies, the support pressure corresponding to the point where the maximum horizontal displacement increases rapidly can be considered as the ultimate support force. Two cases are considered: one where the cohesive strength of the upper and lower strata is similar, and one where it differs significantly. The corresponding soil parameters are shown in (Tables 1 and 2).

**Table 1:** Calculation parameters for the overall failure mode.

Object	E0 (MPa)	*φ* (°)	*c* (kPa)	*γ*_*sat*_ (kN/m^3^)
Upper soft soil	30	20	5	20
Lower hard soil	40	30	15	20
Lining	3.5 × 10^4^	–	–	–

**Table 2:** Calculation parameters for the partial failure mode.

Object	E0 (MPa)	*φ* (°)	*c* (kPa)	*γ*_*sat*_ (kN/m^3^)
Upper soft soil	30	20	5	20
Lower hard soil	100	35	50	20
Lining	3.5 × 10^4^	–	–	–

#### Numerical analysis under overall failure mode

**Fig. 9:**
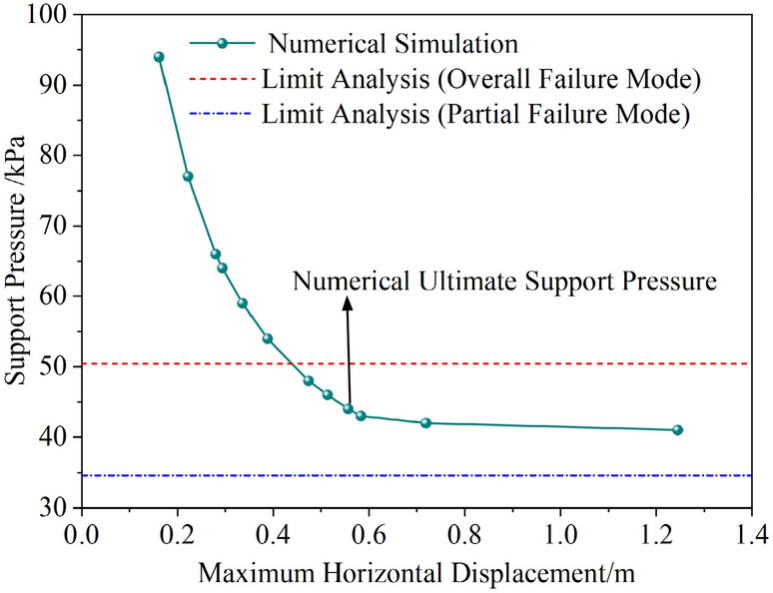
Variation of maximum horizontal displacement with tunnel face support pressure.

**Fig. 10:**
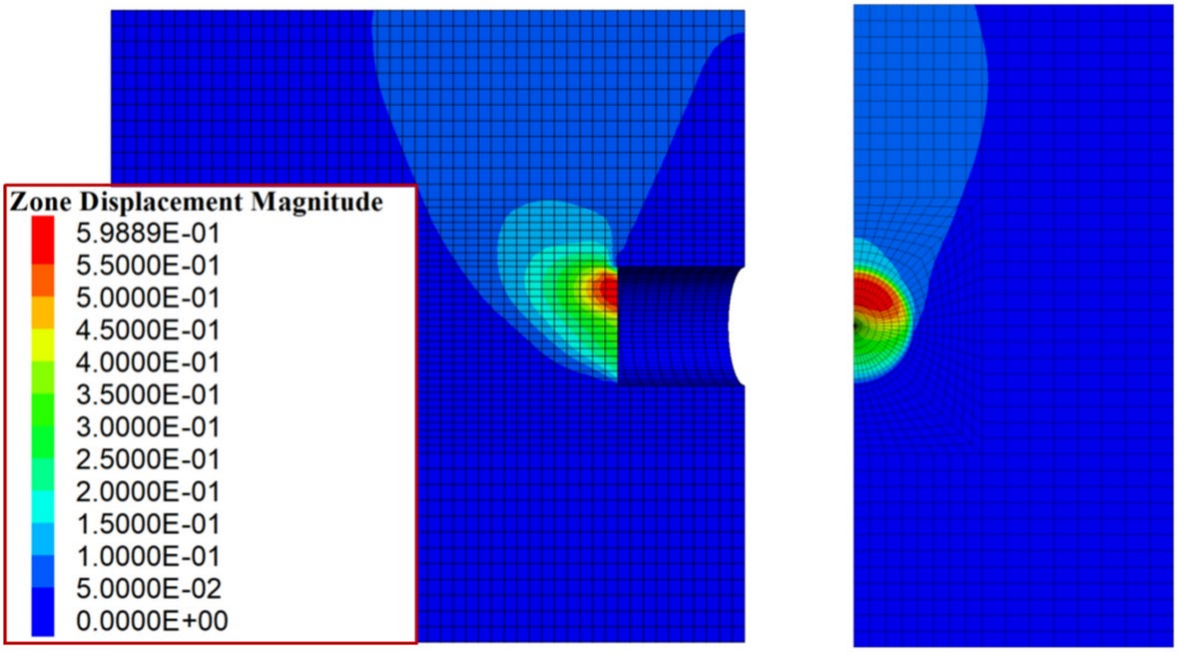
Displacement contour map at ultimate state from numerical simulation.

First, a set of parameters is selected, and the results calculated using the proposed method are as follows: the support pressure under overall failure is 50.43 kPa, and the ultimate support force under partial failure is 34.58 kPa. A subsequent numerical simulation was conducted, and the relationship between the maximum horizontal displacement and the support pressure of the tunnel face is plotted in (Fig. 9). As seen in the figure, the numerical simulation results are in close agreement with the results of the overall failure mode calculated by the proposed method, with a difference of approximately 14%. Figure 10 shows the displacement contour map at the ultimate state from the numerical simulation.

#### Numerical analysis under the partial failure mode

**Fig. 11:**
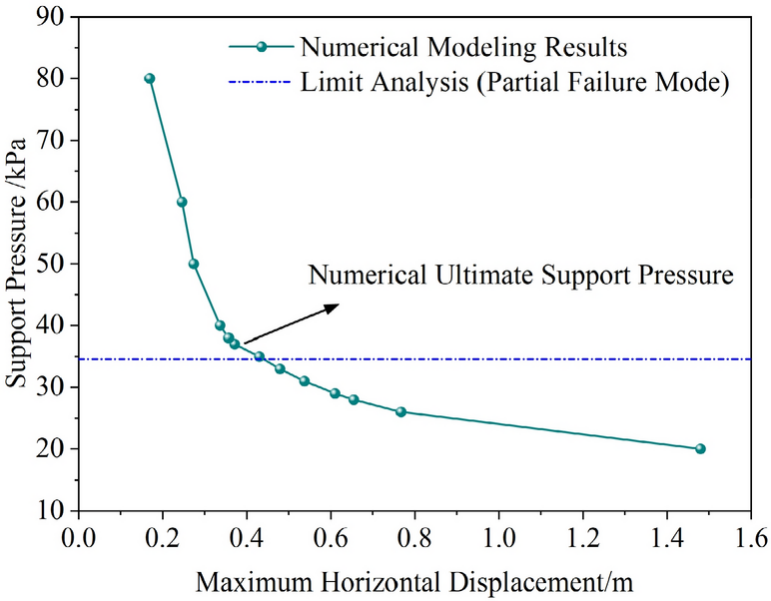
Variation of maximum horizontal displacement with tunnel face support pressure.

**Fig. 12:**
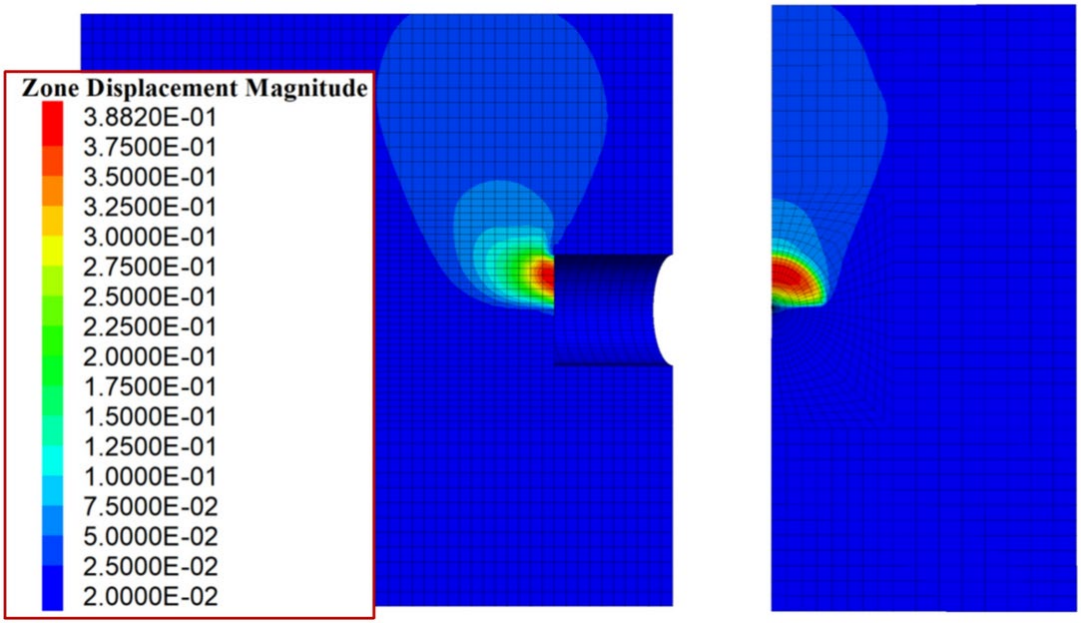
Displacement contour map at ultimate state from numerical simulation.

To calculate the partial failure mode, the strength parameters of the lower hard soil layer are assigned larger values to ensure its self-stability. The results calculated using the proposed method are as follows: the support pressure under the overall failure mode is less than 0 kPa, while the ultimate support pressure under the partial failure mode is 34.58 kPa. A numerical simulation was conducted for comparison, and the relationship between the maximum horizontal displacement of the tunnel face and the support pressure is shown in (Fig. 11). As seen in the figure, the numerical simulation results are very close to the results from the proposed method under the partial failure mode, with a difference of about 7%. Figure 12 shows the displacement contour map for the limit state in the numerical simulation. It can be seen that, under these parameters, the numerical simulation results exhibit a clear partial failure pattern, consistent with the conclusions of the proposed method.

Based on the above analysis, it can be concluded that the difference in calculation accuracy between the proposed method and numerical simulation ranges from 7 to 14%. Overall, this range of difference is acceptable. The accuracy of the proposed method depends on the establishment of the failure mechanism, while the accuracy of the numerical method depends on the grid resolution. In terms of computational efficiency, under the Intel Core i5-12400F 2.50 GHz configuration, when using a discrete number of *n* = 200, the calculation time for a single working condition is 2–3 min. In comparison, the time for a single numerical simulation model is around 30 min under the same configuration. Additionally, to calculate the ultimate support pressure, approximately 10 numerical conditions must be calculated to obtain the curve. Moreover, when tunnel burial depth, diameter, and other parameters change, the numerical simulation method requires re-modelling. Therefore, in terms of computational efficiency, the proposed method outperforms traditional numerical simulation calculations. The disadvantage of the proposed method is that the failure mode cannot be determined in advance, whereas the advantage of numerical simulation is that the failure mode does not need to be predetermined. In the following sections, we will discuss how to address this disadvantage.

## Analysis of the failure mode transition under different parameters

To study the influence of model parameters on failure modes, i.e., how the failure mode changes under different parameters, the concept of a pressure ratio can be defined as follows:

10 $$ \delta = \frac{{\sigma_{t} }}{{\sigma^{\prime}_{t} }} $$

where $\sigma_{t}$ is the critical support pressure for the partial failure mode and where $\sigma^{\prime}_{t}$ is the critical support pressure for the overall failure mode. According to the definition of the pressure ratio, when the pressure ratio is less than 1, partial failure occurs first, and the failure mode of the excavation face is partial failure; conversely, the overall failure mode is partial failure.

On the basis of the previous analysis, the relevant parameters for the critical support pressure of the excavation face include the proportion of soft soil layers, friction angle, cohesion, water level, pore water pressure coefficient, tunnel diameter, and soil saturation. Therefore, the pressure ratio can be expressed as a function of all these influencing parameters:

11 $$ \delta = f(\rho ,D,c_{l} ,c_{u} ,\varphi_{l} ,\varphi_{u} ,H_{w} ,\gamma_{sat,l} ,\gamma_{sat,u} ) $$

where $c_{l}$ and $c_{u}$ are the cohesions of the lower and upper strata, respectively, and where $\gamma_{sat,l}$ and $\gamma_{sat,u}$ are the saturated unit weights of the lower and upper strata, respectively.

To facilitate subsequent analysis, a set of general parameters was established. When a specific parameter was analysed, the other parameters were kept constant according to the general settings. The general parameters are as follows: $\rho$ = 180°, $D$ = 10 m, $c_{l}$ = 20 kPa, $c_{u}$ = 5 kPa, $\varphi_{l}$ = 30°, $\varphi_{u}$ = 20°, $H_{w}$ = 10 m, and $\gamma_{sat,l}$ = $\gamma_{sat,u}$ = 20 kN/m^3^.

**Fig. 13:**
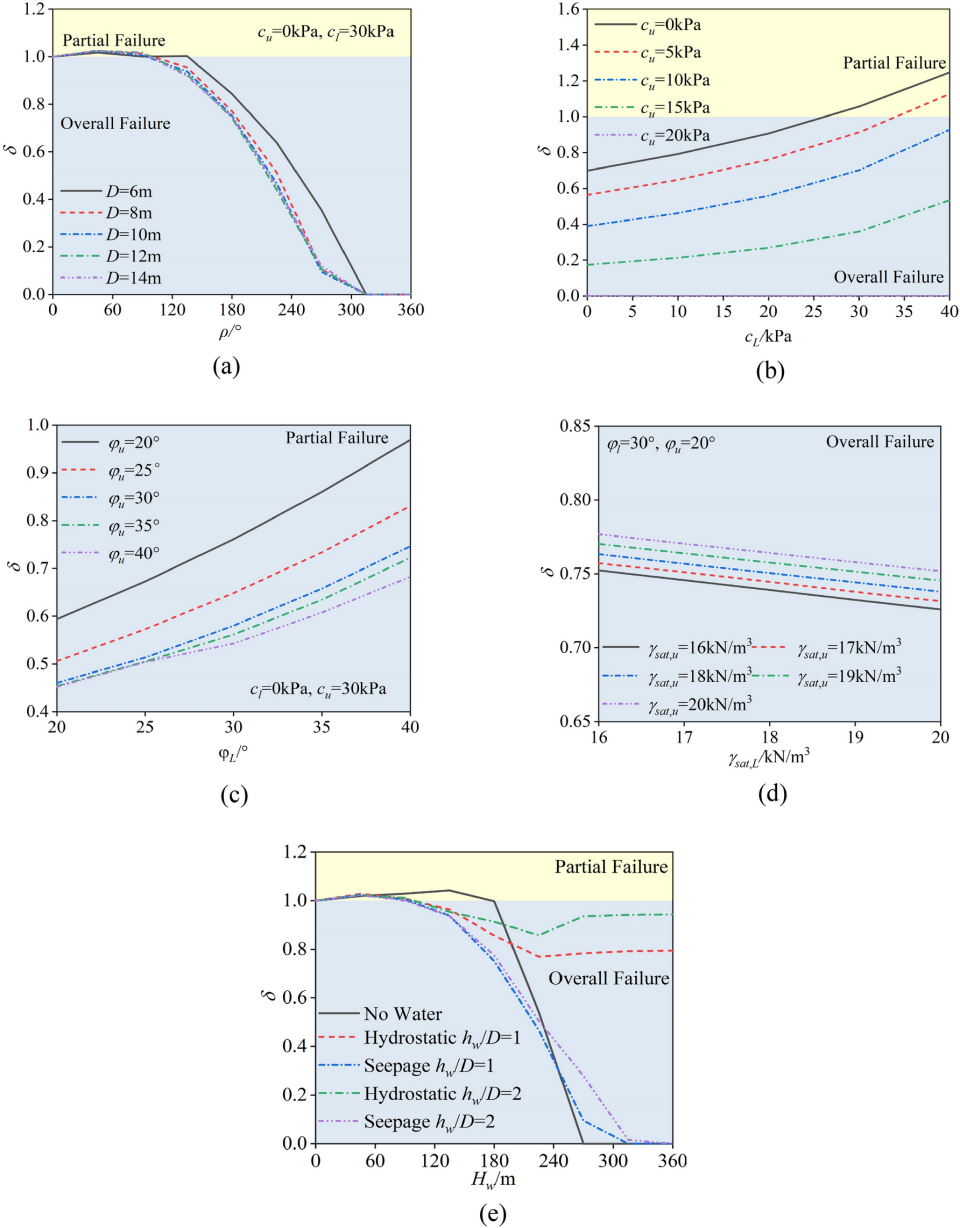
Impact of different parameters on failure modes. (a) $\rho$ and *D*, (b) $c_{l}$ and $c_{u}$, (c) $\varphi_{u}$ and $\varphi_{l}$, (d) $\gamma_{sat,l}$ and $\gamma_{sat,u}$, (e) Water.

Figure 13 illustrates the influence of various parameters on the failure mode:

1. As shown in Fig. 13a, when the tunnel diameter is particularly small (e.g., 6 m), the tunnel face is more prone to local failure. However, when the tunnel diameter is larger, the change in failure mode is almost unaffected by the tunnel diameter. The pressure ratio increases with the increase in the composite angle of the strata, showing a trend of first increasing and then decreasing. At $c_{u}$ = 5 kPa and $c_{l}$ = 20 kPa, tunnels with different diameters all experience local failure when the composite angle of the strata is 60°. As the difference in cohesive forces between the upper and lower strata increases, the range of composite angles for local failure expands further.
2. Figure 13b shows the impact of the cohesive forces $c_{u}$ and $c_{l}$ of the upper and lower layers on the failure mode. $c_{u}$ and $c_{l}$ significantly influence the failure mode, with the difference in the support pressure ratio reaching 1.0 under different $c_{u}$ and $c_{l}$ conditions. The smaller $c_{u}$ is and the larger $c_{l}$ is, the greater the likelihood that partial failure will occur. This effect is nonlinear; as $c_{u}$ increases, the support pressure ratio rapidly increases, whereas an increase in $c_{l}$ significantly reduces the likelihood of partial failure. It is also noteworthy that in the example working conditions, when $c_{u}$ ≥ 20 kPa, the upper layer can achieve self-stability, and at this point, the pressure ratio is 0. Therefore, in composite strata with low cohesive forces in the upper soil layer, attention should be paid to the failure mode, while in cases with higher cohesive forces in the upper soil layer, the possibility of partial failure can be neglected.
3. As seen in Fig. 13c, the impact of $\varphi_{u}$ and $\varphi_{l}$ n the pressure ratio exhibits a more obvious nonlinearity, especially with $\varphi_{u}$. As $\varphi_{u}$ increases, the pressure ratio decreases, and its effect on the pressure ratio gradually weakens. The opposite occurs for $\varphi_{l}$, where the pressure ratio increases as $\varphi_{l}$ increases, and its influence on the pressure ratio becomes greater with higher values of $\varphi_{l}$.This opposite trend may be due to the fact that $\varphi_{u}$ simultaneously influence the failure mechanisms in both the overall and local failure modes. Since the local failure mode is entirely located in the upper stratum, it is more significantly affected by $\varphi_{u}$. For $\varphi_{l}$, it only controls the generation of the failure mechanism under overall failure. Therefore, $\varphi_{u}$ mainly affects the numerator in the pressure ratio calculation, while $\varphi_{l}$ influences the denominator, leading to the opposite impact trend. From a sensitivity perspective, in the entire analysis, the friction angle has the greatest effect on the pressure ratio, with a maximum value of about 0.5, which is smaller than the influence of the composite angle and cohesive force.
4. Figure 13d shows the impact of $\gamma_{sat,l}$ and $\gamma_{sat,u}$ on the failure mode. The influence of $\gamma_{sat,l}$ and $\gamma_{sat,u}$ on the support pressure ratio is relatively small and almost linear. The support pressure ratio has a negative correlation with both $\gamma_{sat,l}$ and $\gamma_{sat,u}$.
5. To illustrate the impact of groundwater on the pressure ratio, calculations were performed for the dry condition, seepage condition, and the hydrostatic condition where no seepage occurs, as shown in (Fig. 13e). It can be observed that in the dry condition, the range of composite angles for local failure is significantly wider, while the influence of seepage is relatively small. Although the pressure ratio in the hydrostatic condition is much greater than in the seepage condition for larger composite angles, the ultimate support pressure is essentially equal to the hydrostatic pressure. This is because the location of the local failure surface is at a higher average height than the overall failure mode, leading to a pressure ratio that is not equal to 1. The influence of groundwater on the failure mode transition is minimal.

In summary, the composite angle of the strata, cohesive forces, and the presence of groundwater are key factors controlling the transition of failure modes. The effect of the friction angle should also not be overlooked, while the influence of unit weight and seepage degree can be ignored.

## Tunnel face stability in water-rich soft-upper hard-lower strata

### Comprehensive critical support pressure considering two failure modes

The analysis in Chapter 4 points out that the method proposed in this paper is more efficient compared to numerical simulation methods. However, the disadvantage of this method is that the failure mode cannot be predetermined. In fact, both the overall and partial failure modes can be simultaneously considered in the calculation of the ultimate support pressure, with the larger result indicating the mode of failure that occurs. Therefore, this paper proposes a comprehensive critical support pressure that accounts for both partial and overall failure modes:

$$ \sigma_{t,g} = \max (\sigma_{t} ,\sigma^{\prime}_{t} ) $$

where $\sigma_{t}$ and $\sigma^{\prime}$ are the critical support pressures corresponding to the overall and partial failure modes, respectively.

On the basis of previous calculations, the comprehensive critical support pressure is related to the relevant parameters $\rho$, *D*, $c_{l}$, $c_{u}$,$\varphi_{l}$, $\varphi_{u}$, $H_{w}$, $\gamma_{sat,l}$, and $\gamma_{sat,u}$. For ease of analysis, the parameters are normalized. Following related studies, the parameter $\gamma_{w} H_{w}$ is used to construct the normalized comprehensive critical support pressure. Thus, the normalized comprehensive critical support pressure can be expressed as:$c_{U}$

$$ \frac{{\sigma_{t,g} }}{{\gamma_{w} H_{w} }} = \psi (\rho ,\frac{D}{{H_{w} }},\frac{{c_{l} }}{{\gamma_{w} H_{w} }},\frac{{c_{u} }}{{\gamma_{w} H_{w} }},\varphi_{l} ,\varphi_{u} ,r_{u} ,\frac{{\gamma_{sat,l} }}{{\gamma_{w} }},\frac{{\gamma_{sat,u} }}{{\gamma_{w} }}) $$

**Fig. 14:**
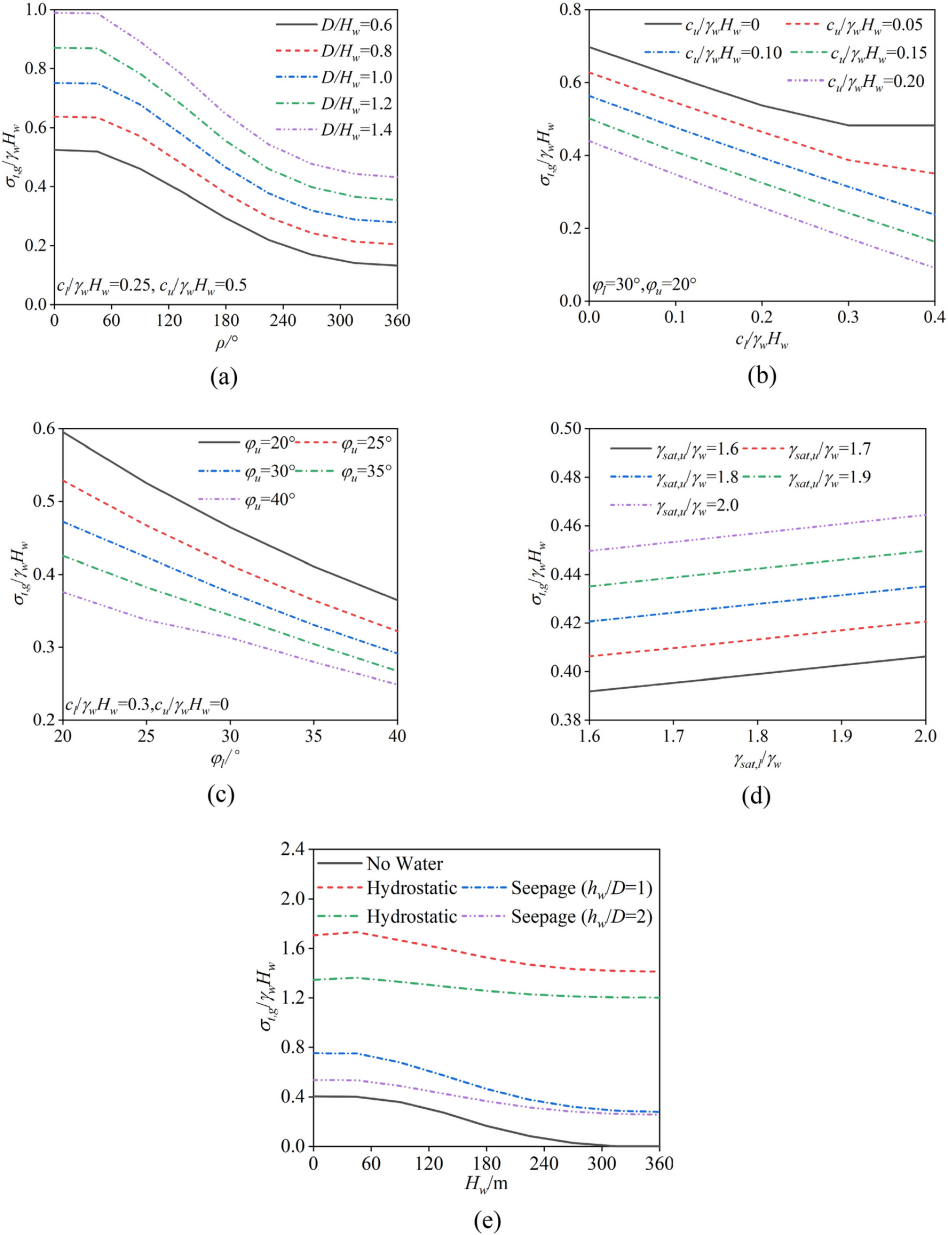
Impact of different parameters on the comprehensive critical support pressure. (**a**) $\rho$ and *D*, (**b**) $c_{l}$ and $c_{u}$, (**c**) $\varphi_{l}$ and $\varphi_{u}$, (**d**) $\gamma_{sat,l}$ and $\gamma_{sat,u}$, (**e**) Water.

On the basis of the variation in different parameters, this paper provides a series of examples of the comprehensive support pressure, as shown in (Fig. 14). After considering both partial failure and overall failure, the variation pattern of the tunnel face critical support pressure is quite complex. For methods that consider only overall failure, if the partial failure critical support pressure exceeds the overall failure critical support pressure, there is a risk of causing partial failure at the tunnel face. Therefore, the support pressure calculated via the method proposed in this paper is more robust for engineering applications.

### Consideration of groundwater effects in tunnel face stability

**Fig. 15:**
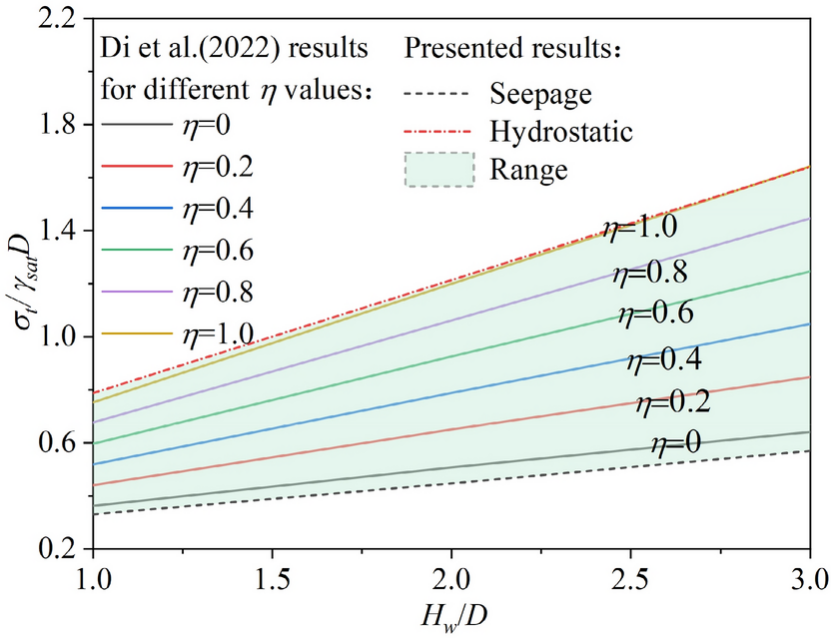
Comparison of proposed range and Di et al.’s results.

In water-rich strata, the influence of groundwater cannot be ignored. Many studies have considered groundwater seepage but typically assume the water pressure at the tunnel face to be zero. However, Di et al.^39^ and Hu et al.^40^ pointed out that the water pressure at the tunnel face is often not zero, though its exact value is difficult to determine. Di et al.^39^ introduced the concept of the excavation face water pressure coefficient to describe the water pressure at the tunnel face, but its value remains difficult to determine. Nevertheless, it is clear that the excavation face water pressure must lie between zero and the hydrostatic pressure. Therefore, both cases can be considered for calculation, and the true ultimate support pressure will lie between these two values. By taking these as the upper and lower limits, a reliable range for the ultimate support pressure can be quickly obtained. Figure 15 shows the comparison between the range proposed in this paper and the results calculated by Di et al.^39^ under different water pressure coefficients. It can be observed that the proposed range fully covers the calculation results under various water pressure coefficients.

## Conclusions

1. Failure Modes in Layered Soils: In horizontally layered soils with soft upper and hard lower strata, tunnel face failure can occur either across the entire cross-section (overall failure mode) or only in the upper layer (partial failure mode). The presence of pore water pressure significantly affects the critical support pressure at which tunnel face failure occurs. Therefore, on the basis of the upper bound theorem of limit analysis and the Mohr‒Coulomb failure criterion, a theoretical calculation method is proposed to determine the critical support pressure for partial or overall failure while considering pore water pressure effects. This method calculates the critical support pressure for partial or overall failure of shield excavation faces in soft upper- and hard lower-layered soils. By comparing with existing studies and conducting numerical simulations for validation, the reliability of this method was confirmed. According to the calculation results, the difference between the results of this method and the numerical simulations ranges from 7 to 14%, demonstrating its advantage in accuracy. The disadvantage lies in the determination of the failure mode.
  The reliability of this proposed method has been validated through a comparison with previous research.
2. The influences of various parameters, such as tunnel diameter, soil cohesion, and pore water pressure, on failure modes were analysed. The results indicate that key factors affecting the failure mode include the proportion of soft soil, tunnel diameter, and the cohesive forces in the upper and lower soil layers. Specifically, when the tunnel diameter is small (e.g., 6 m), partial failure is more likely. Larger diameters, however, have a minimal impact on failure mode. Greater cohesion in the lower soil layer increases the likelihood of partial failure, while the opposite occurs for the upper soil layer. Notably, when the upper layer is sandy and the lower layer is clayey, the probability of partial failure increases significantly. Additionally, when considering the support pressure, neglecting partial failure in the analysis can lead to an underestimation of tunnel face stability. This highlights the importance of considering both overall and partial failure modes in practical applications.
3. To ensure the accuracy of the failure mode, the concept of comprehensive support pressure is proposed, which considers both overall and partial failure modes. Additionally, to account for varying degrees of seepage, the ultimate support pressures calculated under seepage conditions (with excavation face water pressure set to 0) and under hydrostatic conditions are used as the lower and upper bounds, respectively. This approach provides a reasonable range for the ultimate support pressure.

## Data Availability

All data generated or analysed during this study are included in this published article.

## Abbreviations

*C,D*: Buried depth and tunnel diameter
$\rho$: Angle occupied by the hard soil layer
*H*_*w*_: Water level measured from the top of the tunnel
$\theta_{{\text{A}}} ,\;\theta_{{\text{B}}} ,\;\theta_{{\text{G}}}$: Angles between OA, OB, and OG, and the vertical line
$\varphi_{l} ,\;\varphi_{u}$: Friction angles of the lower hard strata and the upper soft strata
${\text{P}}_{j} {,}\;{\text{P}}_{J}^{\prime }$: Discrete points on the excavation profile
*C*_*j*_: The geometric center of radial planes
$\prod_{j}$: Truncation term in seepage calculation
${\text{P}}_{i,j}$: The i-th discrete point on $\prod_{j}$
*S*_*ij*_, Σ_*i*__,__*j*_: Area of triangular and quadrilateral elements
*V*_*i*__,__*j*_: Volume of the triangular element
*W*_*σ*_, *W*_*G*_, *W*_*e*_, *W*_*w*_: Power of support pressure, weight of soil, cohesion, and pore water pressure
$u_{i,j} ,\;u_{j}$: Volume of the triangular element
*σ*_*t*_: Limit support pressure
$c_{l} ,\;c_{u}$: Cohesions of the lower and upper strata
$$\delta$$: Pressure ratio
$\gamma_{sat,L} ,\,\gamma_{sat,U}$: Saturated unit weights of the lower and upper strata
$\sigma_{t} ,\sigma^{\prime}$: Critical support pressures corresponding to the overall and partial failure modes
$\sigma_{t,g}$: Comprehensive critical support pressure
